# Methylation of CpG 5962 in L1 of the human papillomavirus 16 genome as a potential predictive marker for viral persistence: A prospective large cohort study using cervical swab samples

**DOI:** 10.1002/cam4.2771

**Published:** 2019-12-19

**Authors:** Jasmin Fertey, Jörg Hagmann, Hans‐Joachim Ruscheweyh, Christian Munk, Susanne Kjaer, Daniel Huson, Juliane Haedicke‐Jarboui, Frank Stubenrauch, Thomas Iftner

**Affiliations:** ^1^ Medical Virology Institute of Medical Virology University Hospital of Tuebingen Tuebingen Germany; ^2^ Department of Molecular Biology Max Planck Institute for Developmental Biology Tuebingen Germany; ^3^ Centre for Bioinformatics Tuebingen University Tuebingen Germany; ^4^ Unit of Virus, Lifestyle and Genes Danish Cancer Society Research Center Copenhagen Denmark

**Keywords:** Biomarker, DNA Methylation, HPV16, Persistence

## Abstract

Several studies have demonstrated that the viral genome can be methylated by the host cell during progression from persistent infection to cervical cancer. The aim of this study was to investigate whether methylation at a specific site could predict the development of viral persistence and whether viral load shows a correlation with specific methylation patterns. HPV16‐positive samples from women aged 20–29 years (n = 99) with a follow‐up time of 13 years, were included from a Danish cohort comprising 11 088 women. Viral load was measured by real‐time PCR and methylation status was determined for 39 CpG sites in the upstream regulatory region (URR), *E6/E7,* and *L1* region of HPV16 by next‐generation sequencing. Participants were divided into two groups according to whether they were persistently (≥ 24 months) or transiently HPV16 infected. The general methylation status was significantly different between women with a persistent and women with a transient infection outcome (*P* = .025). One site located in *L1* (nt. 5962) was statistically significantly (*P* = .00048) different in the methylation status after correction using the Holm‐Sidak method (alpha = 0.05). Correlation analyses of samples from HPV16 persistently infected women suggest that methylation is higher although viral load is lower. This study indicates that methylation at position 5962 of the HPV16 genome within the *L1* gene might be a predictive marker for the development of a persistent HPV16 infection.

## Graphical abstract

Persistent HPV16 infections are the main risk factor for the development of cancer of the cervix uteri. Currently, no biomarkers are available to determine the probability for a persistent HPV16 infection after a single HPV test. This study indicates that DNA methylation at position 5962 in the HPV16 L1 gene might be a predictive marker for a persistent HPV16 infection.

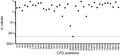

## 1 INTRODUCTION

A persistent infection with high risk (HR) types of human papillomaviruses (HPV) is a necessary precondition for cervical cancer.^1^ Although the latest generation of vaccines against HPV infections is highly promising, a large number of women already persistently infected will continue to rely on screening for cervical cancer precursors in the upcoming decades. HPV prevalence peaks in women aged between 18 and 25 years and sharply declines with increasing age. While the vast majority of women acquire a HR HPV infection during their lifetime, only a minority of infections persist and progress to cervical cancer or precancerous lesions, defined as high‐grade cervical intraepithelial neoplasia grade 3 (CIN3+).^2^ In a time span of 2‐3 decades, approximately a third of untreated CIN3 lesions become invasive and progress to cervical cancer.^3^ The transition from a productive to a transforming infection from CIN1 to CIN3 lesions is characterized by a substantial change in HPV gene expression, from high‐level structural gene (L1 and L2) expression and low oncogene (E6 and E7) expression to high expression of oncogenes that interfere with cellular apoptosis and cell cycle regulation and diminished expression of structural proteins.^4^ Altered viral gene expression has been linked to methylation of the viral genome for several DNA viruses including Kaposi Sarcoma Herpesvirus, Epstein‐Barr Virus, Simian Virus 40 and Adenoviruses (reviewed by 5). DNA methylation, which is regulated by DNA methyltransferases (DNMTs) is also a common mechanism in mammalian cells to silence genes; and aberrant methylation patterns have been detected in many different promoters controlling tumor suppressor genes in cancer cells and in cancer‐derived cell lines.^6^, ^7^, ^8^, ^9^ Recent findings identified a second class of enzymes involved in demethylation, the Ten‐eleven‐translocation proteins (TETs) that led to the conclusion that gene silencing by methylation is a reversible state.^10^ It has been demonstrated that the papillomavirus genome is de novo methylated by DNMTs of the host cell that also play a role in innate immunity.^5^, ^7^ Therefore methylation of the viral genome might be a novel mechanism by which the host cell regulates viral gene expression and thereby HPV pathogenicity.

For several papillomaviruses it was shown, that during their normal lifecycle upon differentiation of the host cell, the viral URR becomes hypomethylated highlighting the dynamics of methylation‐dependent gene expression regulation.^9^, ^11^, ^12^, ^13^ Therefore several studies have analyzed the correlation between HPV associated lesions and methylation of the viral genome with the common observation that methylation reaches the highest degree in cancer samples.^6^, ^14^, ^15^, ^16^

Experiments in tissue culture indicate that integration of the viral genome into the host chromosome may lead to an alteration in methylation patterns on the viral or host genome depending on the type of integration event.^8^, ^9^ Integration of the viral DNA into the host genome has been demonstrated to increase during persistence or carcinogenesis.^17^, ^18^, ^19^

Recent studies in HPV16‐infected women demonstrated that the levels of methylation slowly increase with enduring HPV persistence, with the diagnosis of cervical cancer and with age.^16^, ^20^ Most of the studies focused on samples derived from precancerous lesions and cancer and only a few studies have been conducted using a prospective study design. Furthermore, very few studies have been conducted combining Methylation status and viral load as possible predictors of future cervical disease.^20^, ^21^ With the samples used in this prospective cohort study it was possible to investigate the methylation status as well as the viral load years before CIN development. Due to the personal identification number that is being used in the pathology registry in Denmark, data on health status can be easily followed up and assigned to each patient years after the sample was taken. This unique feature allowed us to analyze the HPV16 genome methylation at an early time point of infection.

## 2 MATERIAL AND METHODS

### 2.1 Study population

The Danish HPV cohort has previously been described.^17^, ^22^ In brief, women were randomly selected from the general female population of Copenhagen, Denmark, using the unique 10‐digit personal identification number, which is universally used. All women had cervical swabs taken at enrollment and at approximately 2 years later.

The existence of nationwide registers and the unique personal identification numbers ensure valid linkage between the pathology registry, and facilitate follow‐up studies with virtually no loss to follow‐up. The women within this study cohort were linked to the Pathology Data Bank and followed up until 2014 identifying all cervical lesions and diagnostic or treatment procedures. As HPV DNA testing took place several years after the study examinations were performed, the women were unaware of their HPV test results; and these results had no influence on clinical management of these women. All women were between the ages of 20 and 29 at the time the first samples were taken. The study was approved by the national Scientific Ethical Committee and the national Data Protection Board.

### 2.2 Study design

In total 101 samples from women with single HPV16 infections from the enrollment examination were included and methylation analysis was performed. Two samples failed to amplify and were excluded from the study leaving 99 women within this study. To investigate whether methylation of the viral genome determines the outcome of an infection, DNA from cervical swabs was extracted, bisulfite treated and analyzed by next‐generation sequencing on a Roche 454 platform. We chose next‐generation sequencing for methylation analysis after bisulfite treatment to get quantitative data for each methylated CpG site, because cervical samples may contain molecules with mixed methylation status at the same CpG position. As DNA content and quality of the cervical swab samples was highly variable, not all regions could be amplified with the same efficiency after bisulfite treatment. After sequencing, the reads were sorted according to the respective tags to receive one pool per patient, nonfunctional sequences like primer or adapter sequences were removed and the reads were filtered according to their quality. We initially chose 50 CpG sites in the following regions for analysis: Enhancer/URR, E6/E7 and L1. However, multiple CpG sites within the L1‐URR fragment yielded no signal or not enough information after quality filtering of the dataset. Therefore, we decided to only analyze those regions that match high‐quality standards and contain sufficient information for statistical analysis, reducing the number of CpG sites further analyzed to 39.

**Figure 1:**
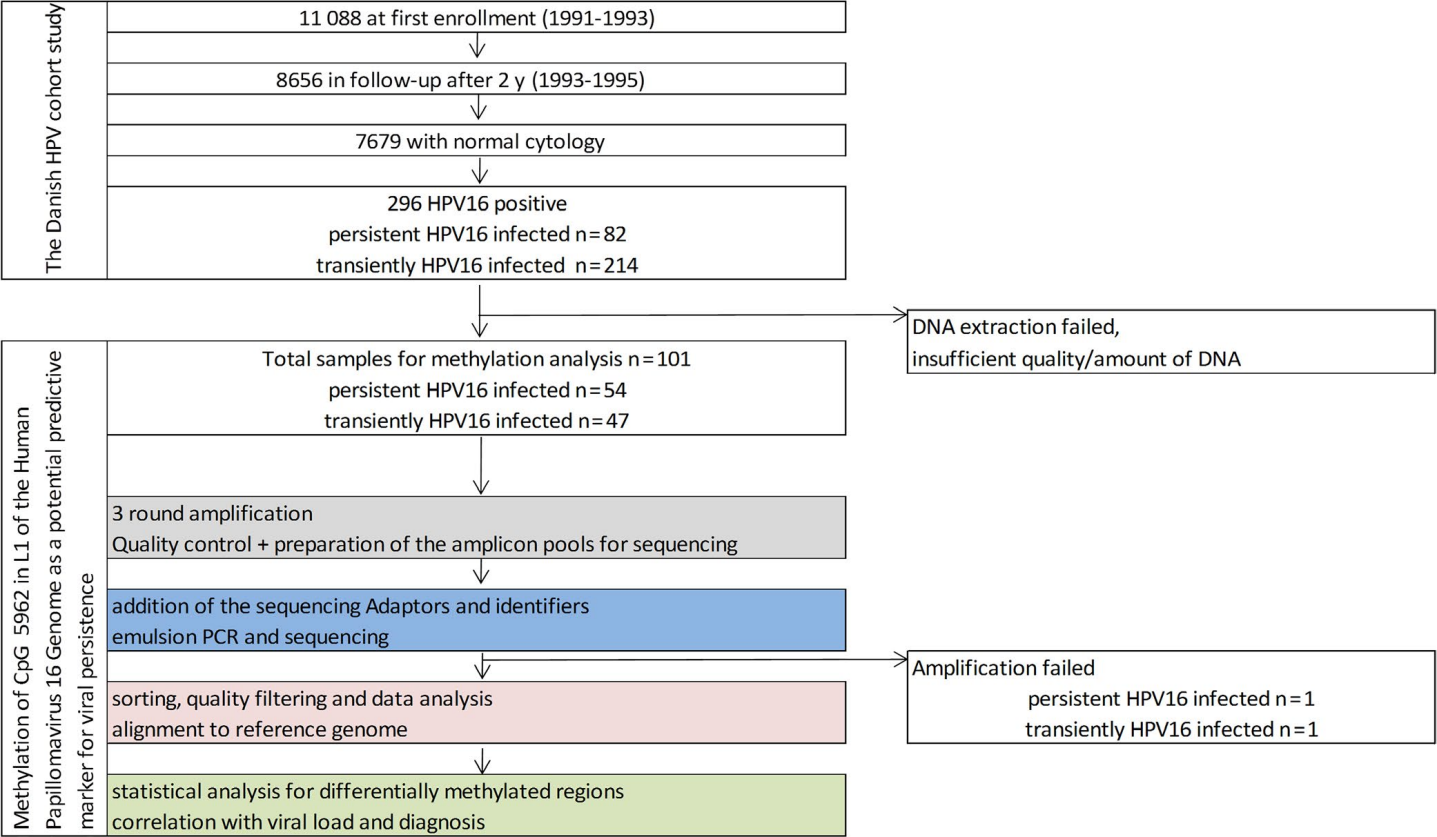
Schematic overview of the study population. Overview of study design of the Danish HPV cohort study (modified from 32). For methylation analysis 101 HPV‐positive samples were selected, n = 99 samples have been finally included. Of these 99 samples, 53 remained HPV16‐positive after 2 years and were therefore regarded as persistently infected. 46 were negative for HPV16 after 2 years and were therefore regarded as transiently infected

Women were included in the analysis who were also HPV16‐positive at the second examination, that is, women, who were HPV16‐positive at two time points with two years in between, were considered to have a persistent HPV16 infection (n = 53), whereas women who were HPV16 negative at the second examination were considered to have cleared their infection (n = 46) (Figure 1). Finally, via linkage to the Pathology Data Bank we were able to determine the outcome of a persistent HPV16 infection. This way, we also assessed the role of specific methylation sites for the development of cervical disease of CIN2+ cases occurring up to 12 years following the second examination.

### 2.3 HPV testing

Residual LBC samples were tested using the Hybrid Capture 2 (HC2) HPV test (Qiagen) using the HR probe set in the central molecular testing laboratory (UKT, Tuebingen) as previously described.^23^ HPV genotyping was carried out using the INNO‐LiPA HPV Genotyping v2 test as previously described.^24^

### 2.4 Cell culture

SiHa (low‐copy HPV16) and Caski (high‐copy HPV16) cell lines were cultured in DMEM (Life Technologies) supplemented with 10% FCS (PAA) and gentamycin (Life Technologies).

### 2.5 DNA isolation and bisulfite treatment

DNA was isolated from cervical swabs or cell cultures using the QiaSymphony robotic system (Qiagen) and the QiaSymphony Virus/Bacteria kit (Qiagen) with an 800 µl extraction protocol according to manufacturer’s instructions. Extracted DNA was subjected to bisulfite treatment using the Epitect DNA Methylation Kit (Qiagen). In brief, 200–500 ng of DNA (depending on the DNA concentration of the sample) were converted according to the low concentrated samples‐protocol of the manufacturer. During bisulfite treatment unmethylated cytosines (C) are delaminated, which are then converted into uracil (U), and replaced with thymine (T) during PCR amplification; methylated C nucleotides are protected from conversion and remain unmodified.

### 2.6 Primer design and PCR

Primers flanking the CpG sites of interest were designed using an in silico bisulfite converted HPV16 sequence (Human papillomavirus type 16 clone 114/K, complete genome 7,906 bp circular DNA EU118173.1 GI:157087542). 4 µl of the bisulfite‐treated sample was used for subsequent preamplification using six primer pairs, generating four larger amplicons. These served as template for the subsequent amplification that generated 12 amplicons, the primers are listed in Table S1 without M13 linker sequence, which was added to attach the identifier for next‐generation sequencing. All segments were amplified by OneTaq DNA polymerase (NEB). The PCR for each method was carried out in 50 µl reaction volumes in an MJ research Thermal Cycler in a 96 well format with the following parameters: initial denaturation at 94°C for 5 minutes, followed by 45 cycles of 94°C for 30 seconds, 50°C for 1 minute, and 72°C for 1 minute with a final extension at 72°C for 10 minutes. PCR products were run on 1.5% agarose gel electrophoresis to check efficient amplification.

### 2.7 Quantitative realtime PCR

Quantitative RealTime PCR was performed for viral load and integration status measurements as described.^17^ Predesigned primers (QT00203763, Qiagen) were used for detection of the single copy gene *IFNB1* for normalization. The HPV16 *E2* and *E6* primers have previously been described.^25^ PCR was performed in a final volume of 20 μl containing 1 × Light Cycler 480 SYBR green I Master Mix (Roche Diagnostics), 0.3 μM primers and 5 μl of DNA.

### 2.8 PCR purification and 454 Nextgen‐sequencing

In a first step, multiplex identifiers (MIDs) for library preparation were added in an additional PCR reaction described above using primers that carry an M13 sequence to create identical ends for the Amplicon pools. The sequence is described in Table S1. PCR products of each patient were pooled and purified using QiaQuick nucleotide removal kit (Qiagen) for subsequent 454 sequencing. 454 sequencing was carried out on the Titanium platform (Roche/454 Life Sciences) by Eurofins/MWG Operon, Erlangen Germany.

### 2.9 Data analysis

The amplicons were sorted by barcodes (MIDs) to set up 101 libraries for analysis. All sequences were quality trimmed followed by cutting off nucleotides with low quality (<20, sliding window 7).Short reads, remaining fragments of MID and primer sequences were removed after demultiplexing. The reads were collapsed, so that no identical reads were present. The resulting reads were aligned to the reference sequence and percentage of methylation was calculated.^26^ As reference sequence a consensus sequence of 22 HPV16 variants was used. The consensus sequence was generated with MegAlign (SeqMan NGen^®^) using the following sequences (Genebank Accession Numbers): EU118173.1, AY686579.1, AY686581.1, AY686583.1, AY686582.1, EU918764.1, AF534061.1, K02718.1, NC_001526.2, HM057182.1, AF472508.1, AF472509.1, AF536179.1, AF536180.1, AF402678.1, FJ610150.1, FJ610146.1, FJ610151.1, FJ610149.1, AF125673.1, AY686584.1, AY686580.1.

### 2.10 Statistical analysis

For analysis of viral load as risk factor for persistence, we compared transiently HPV16‐infected women with persistently HPV16‐infected women using cervical samples taken at the initial examination at enrollment. The Mann‐Whitney test was used to test for significant differences. All statistical tests were conducted at the 5% two‐sided significance level. For multiple comparisons the correction was performed using the Holm‐Sidak method^27^ with alpha = 0.05 assuming that all rows are sampled from populations with the same scatter (SD). Statistical tests and multiple testing were performed using GraphPad Prism 6^®^ (GraphPad Software). Graphs were generated using GraphPad Prism 6^®^. Heatmaps were generated using the web‐based application “CIMminer” (http://discover.nci.nih.gov/cimminer/home.do).

## 3 RESULTS

### 3.1 Methylation as predictor of HPV16 infection outcome

**Figure 2:**
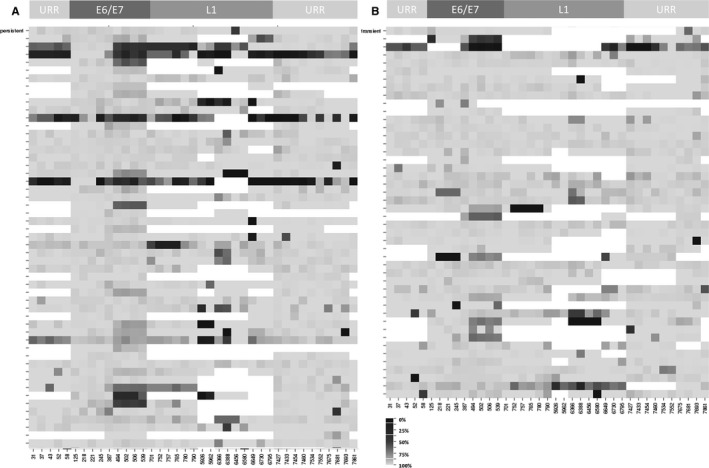
Methylation status distribution for each CpG site. A, Methylation profiles for samples from persistently infected women. Each row represents one prospective persistent patient sample. The HPV16 genome with each CpG site is listed horizontally at the bottom. The top panel indicates the respective region of the viral genome. The level of site‐specific methylation is shaded according to the scale bar from 0% – 100%. White indicates that the site did not exist in the given sample or failed to amplify. B, Methylation profiles for samples from transiently infected women

Figure 2A shows the persistent samples, whereas Figure 2B displays the transient samples. White regions indicate missing data, black indicates 100% methylation, whereas gray shades indicate the respective percentage of methylation. To control for the efficiency of the bisulfite treatment and the PCR reactions, DNA from the cervical carcinoma cell lines SiHa and Caski served as positive controls. SiHa cells are generally regarded as low methylated, because they harbor only 1‐2 HPV16 copies, which are highly transcriptionally active. Caski cells display a higher overall methylation of the viral genome, because they contain up to 600 integrated HPV16 copies of which most of them are transcriptionally silenced by DNA methylation.^6^, ^28^, ^29^ In agreement with this, SiHa cells showed a significantly lower methylation level than Caski cells except for the *L1* region (Figure S1B).

**Figure 3:**
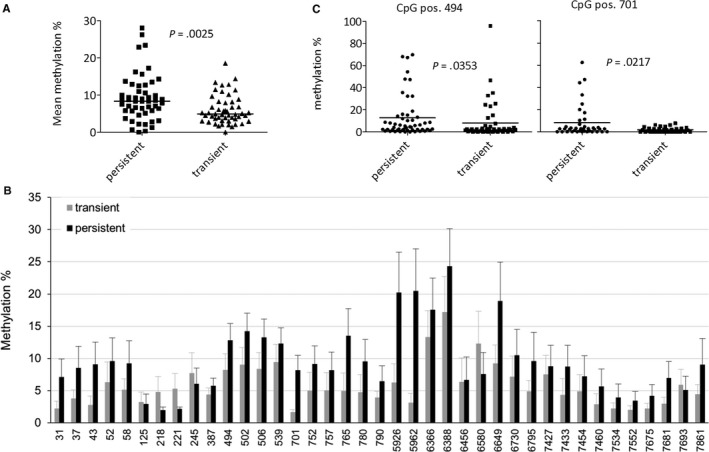
The methylation status differs between samples with persistent and transient infection outcome before correction for multiple testing. A, Overall comparison of the quantities of methylation between transiently and persistently infected women. For this, results were grouped according to the patient’s future infection outcome and the mean of the methylation at every CpG was calculated, (each dot represents the mean value of one CpG position). The *P* value was calculated using the nonparametric, two‐tailed Mann‐Whitney test. B, Comparison of methylation quantities at each CpG site between transiently and persistently infected women. The mean of the methylation amount at each CpG was calculated, (each bar represents the mean value). SEMs are indicated. C, Comparison of methylation quantities at CpG sites 494 and 701 between transiently and persistently infected women. The mean of the methylation at every CpG was calculated, and a Mann‐Whitney test was applied to test for significant difference between the two groups for each CpG. The only sites that showed statistical significance (*P* < .05) were CpGs 494 and 701

To investigate the methylation pattern in patient samples with known infection outcome, women with confirmed HPV16 infections (n = 99) were selected and only the samples from the initial examination were investigated in this study. The patients were grouped according to their future infection outcome and the average methylation of 39 sites was analyzed using the Mann‐Whitney test. The mean methylation status was significantly different between a persistent and a transient infection outcome (*P* = .025) (Figure 3A). The mean methylation of the particular CpG sites showed a higher methylation in the majority of the CpG positions for the samples that will develop a persistent infection. In detail, the mean methylation status of the individual CpG sites displayed a highly variable distribution with the highest methylation levels in the L1 region (Figure 3B). The two patient groups were further analyzed for specific CpG positions. Since testing for normal distribution (Shapiro‐Wilk *P* < .05) failed, the nonparametric Mann‐Whitney U‐test was applied. Before correction for multiple testing, there were two sites below *P* = .05 that segregated samples that will develop a persistent infection from those who will clear the virus and will therefore be transiently infected. One site is located in *E6* (bp 494, *P* = .016), the other site in *E7* (bp 701, *P* = .022) (Figure 3C).

**Figure 4:**
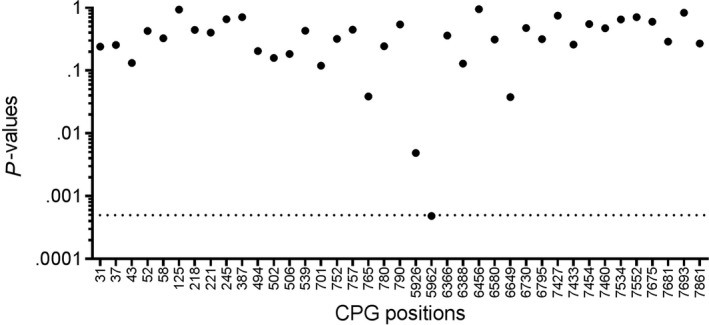
Methylation differs between samples with persistent and transient infection outcome after correction for multiple testing. The samples were grouped according to their future infection outcome and a multiple comparison was applied: persistent samples were compared with transient samples using one pooled SD. The correction for multiple testing was performed using the Holm‐Sidak method with alpha = 0.05. The dashed line indicates the threshold for statistical significance. Dots on and below this line are considered statistically significant

To correct for multiple testing at the methylated CpG positions, a multiple comparison was applied to the data and correction for multiple testing was performed using the Holm‐Sidak method with alpha = 0.05, assuming that all rows are samples from populations with the same scatter. An unpaired t test for each row was computed, using one pooled standard deviation (SD). The results are shown in Table 1. Only one CpG site remained highly significant after correction for multiple testing and is therefore considered differentially methylated in cervical samples of women that develop a persistent HPV16 infection and that clear the infection (Figure 4). The site is located in L1 (bp 5962, *P* = .00048), a region, which has been proposed by several studies to be a prognostic marker for cancer development.^30^, ^31^

**Table 1:** Detailed overview of the CpGs analyzed by multiple comparison. The correction for multiple testing was performed using the Holm‐Sidak method with alpha = 0.05

CpG position	Significant?	P value	Mean1	Mean2	Difference	SE of Difference	T ratio
31		0.2407	7.1256	2.2222	4.9034	4.1785	1.1735
37		0.2567	8.5154	3.7750	4.7404	4.1785	1.1345
43		0.1322	9.0564	2.7639	6.2925	4.1785	1.5059
52		0.4293	9.6000	6.2972	3.3028	4.1785	0.7904
58		0.3275	9.2333	5.1417	4.0917	4.1785	0.9792
125		0.9353	2.9154	3.2159	‐0.3005	3.7032	0.0812
218		0.4440	1.9808	4.8159	‐2.8351	3.7032	0.7656
221		0.4020	2.1731	5.2773	‐3.1042	3.7032	0.8382
245		0.6570	6.0577	7.7023	‐1.6446	3.7032	0.4441
387		0.7131	5.7377	4.3978	1.3399	3.6431	0.3678
494		0.2045	12.8321	8.2087	4.6234	3.6431	1.2691
502		0.1594	14.2019	9.0444	5.1574	3.6647	1.4073
506		0.1836	13.2547	8.3800	4.8747	3.6647	1.3302
539		0.4320	12.3000	9.4200	2.8800	3.6647	0.7859
701		0.1196	8.1475	1.6806	6.4669	4.1533	1.5571
752		0.3198	9.1075	4.9750	4.1325	4.1533	0.9950
757		0.4501	8.1875	5.0500	3.1375	4.1533	0.7554
765		0.0387	13.5350	4.9444	8.5906	4.1533	2.0684
780		0.2452	9.5550	4.7278	4.8272	4.1533	1.1623
790		0.5449	6.4650	3.9500	2.5150	4.1533	0.6055
5926		0.0049	20.2516	6.2348	14.0168	4.9753	2.8173
5962	yes	0.0005	20.5000	3.1217	17.3783	4.9753	3.4929
6366		0.3628	17.5688	13.3107	4.2580	4.6783	0.9102
6388		0.1293	24.3156	17.2179	7.0978	4.6783	1.5172
6456		0.9531	6.6469	6.3714	0.2754	4.6783	0.0589
6580		0.3128	7.5625	12.2857	‐4.7232	4.6783	1.0096
6649		0.0379	18.9343	9.2115	9.7228	4.6807	2.0772
6730		0.4784	10.4914	7.1731	3.3184	4.6807	0.7089
6795		0.3180	9.5629	4.8885	4.6744	4.6807	0.9986
7427		0.7509	8.7620	7.5359	1.2261	3.8623	0.3175
7433		0.2592	8.7100	4.3513	4.3587	3.8623	1.1285
7454		0.5515	7.1980	4.8974	2.3006	3.8623	0.5956
7460		0.4745	5.6600	2.8974	2.7626	3.8623	0.7153
7534		0.6534	3.9320	2.1974	1.7346	3.8623	0.4491
7552		0.7081	3.4460	2.0000	1.4460	3.8623	0.3744
7675		0.5989	4.2063	2.2093	1.9970	3.7961	0.5261
7681		0.2896	6.9833	2.9628	4.0205	3.7961	1.0591
7693		0.8320	5.0854	5.8907	‐0.8053	3.7961	0.2121
7861		0.2702	9.0359	4.4278	4.6081	4.1785	1.1028

Note

Mean1 is referring to the group of women persistently infected; Mean2 is referring to the group of women transiently infected; Difference is the difference between Mean2 and Mean1; T ratio is Mean1 plus Mean2 divided by the SE (standard error) of the difference.

We also analyzed the methylation in persistently infected samples regarding a predictive association with CIN2 development. CIN 2+ encompasses CIN 2, CIN 3, adenocarcinoma in situ (AIS), and cancer. The histological diagnosis has been described in detail.^17^ Of the 53 women with a persistent HPV16 infection, 28 went on to develop a CIN2+ during follow‐up, whereas 25 remained normal. No statistically significant association between methylation and CIN2+‐development was found, indicating that at this early time point no specific methylation pattern predicted development of severe disease (data not shown).

### 3.2 Determination of HPV16 viral load

As previously described 32 out of the 11 088 women aged 20–29 years, 82 were diagnosed persistently infected with HPV16 (Figure 1). Viral load for these samples has previously been analyzed for the baseline and the follow‐up examination (Figure S1A).^17^ Viral loads at enrollment ranged from one to over 1000 viral DNA copies per cell for both the persistent and the transient samples. The mean viral load was significantly higher in cervical samples from transiently HPV16‐infected women (*P* = .006).

### 3.3 Correlation of viral load with methylation of the HPV16 genome

Generally, samples that will develop a persistent infection displayed a lower viral load (Figure S1A) and a higher methylation of the genome (Figure 3B), whereas samples that remain transiently infected showed a higher viral load and a lower methylation status of the genome. Correlation of methylation and viral load was investigated as described before. Five sites were identified that showed a significant result (Figure S2): One in *E6* (bp 494, *P* = .0202), two in *E7* (bp 502, *P* = .0035; bp 539, *P* = .0151), and two in *L1* (bp 5616, *P* = .0103; bp 7033, *P* = .0089). The strongest correlation was observed for site 5616 with a correlation coefficient of r = 0.4407. These data indicate that there is a weak to moderate positive correlation between methylation and viral load.

## 4 DISCUSSION

In this prospective cohort study, we investigated viral DNA methylation of HPV16 at 39 CpG sites in the URR, *E6/E7,* and *L1* region. We used samples that were taken before diagnosis from a cohort of HPV16‐infected women within the prospective Danish HPV Cohort from Copenhagen, Denmark.^22^ Our observations show that it might be possible to predict a persistent infection as a risk factor for disease progression after a single test at a given time point.

We identified two CpG sites in *E6/E7* that have statistically significant differences in methylation levels among HPV16‐infected women before correction for multiple comparisons. Methylation levels were generally higher in samples from women that became persistently infected than in samples from women with a transient infection outcome.

The identified CpGs are not completely consistent with the results from other studies. On one hand, this might be due to the sample material as most of the studies conducted so far focused on cervical cancer or precancer samples and not on cervical smears before cancer diagnosis as in this study. On the other hand, this difference might be based on the fact that these studies had a nonprospective design. Most of these reports describe a hypermethylation of the *L1* and *L2* region in tumor samples.^16^, ^30^, ^33^, ^34^, ^35^, ^36^, ^37^, ^38^, ^39^ The *E6/E7* region has not been in the focus of most studies conducted so far. In productive infections, E6 and E7 are always expressed but at relatively low levels. During carcinogenic progression, transcription of E6 and E7 increases often due to disruption of the E2 ORF after integration of the viral genome. Only the studies, which investigated whole HPV genome methylation provide data for this region^15^, ^20^ and found no significant differences. Other reports mainly focused on the URR but found inconsistent results. Several studies showed elevated methylation levels which were associated with CIN development,^14^, ^15^, ^16^, ^40^, ^41^ whereas others had opposite findings.^6^, ^42^, ^43^, ^44^ Results from a prospective cohort study described the association between HPV16 DNA methylation and cervical disease using serially taken samples analyzing 67 CpG sites distributed over the HPV16 genome.^35^ In HPV16‐positive samples that were taken before onset of disease or of HPV clearance, a CpG site in *L2* (position 4261) showed increased methylation associated with development of CIN3. Others demonstrated significantly different methylated positions in the L1‐region as well, but several reports identified CpGs between 5600 and 5617 as targets 45, 46, 47 or CpGs 6368, 6405, and 6443.^48^, ^49^ The CpG position we identified after correction for multiple testing (bp 5962) differs from the site that was identified in these studies. The major reason for this difference might be that the other studies used cancer or precancer samples, while we focused on prospective samples before establishment of viral persistence or development of cytological abnormalities. It was further demonstrated that increased methylation at CpG sites in the *E2*, *L2*, and *L1* region was associated with a risk of CIN3 compared to women who cleared the infection.^35^ Later Mirabello et al confirmed their findings in a larger number of samples and showed an increased methylation in *L2, L1* and *E2/E4* regions among cases with CIN2+.^20^ We could not identify a statistically significant association between methylation within these regions and CIN2+‐development in our prospective study, which suggests, that specific methylation patterns might not exist at the first time of HPV16 detection and probably develop later during progression of the infection.

Mirabello et al demonstrated that viral load did not affect methylation levels and that women older than the median age of 28 years tended to have higher methylation levels compared to women younger than 28 years.^20^ We here analyzed samples of the young Danish cohort, which involves women 20–29 years of age at the time of enrollment. Therefore, we cannot make any statement whether methylation increases with age. At this time point, it is unclear whether the patterns of HPV16 methylation we observed would be the same for older women. Another study used laser micro dissection to capture different layers of the epithelium within lesions of HPV16‐positive patients. Investigations of the methylation status of the HPV16 URR revealed dynamic changes in the methylation status in the context of the viral life cycle, demonstrating that the methylation status of the HPV16 genome is highly dynamic upon differentiation of the host cell.^13^ Furthermore, the authors showed that neoplastic transformation was associated with methylation of two distinct CpG sites within the distal E2 binding site 1 (E2BS1) leading to URR hyperactivation. Other recent studies revealed that hypermethylation of E2BS3 and E2BS4 together with high viral load was associated with a worse cancer‐specific survival rate in vulvar but not in vaginal carcinoma.^21^ Taken together, a positive correlation of methylation and viral load might determine the outcome of a HPV infection.

One limitation of this study is the number of missing sequences particularly within the *L1* region. This lack of data can only be compensated by assessing our data in a validation cohort. This, however, would require a large prospective patient cohort study with an extended follow‐up period, which is out of scope of this study.

In summary, results from other prospective studies are not fully consistent with the CpG methylation patterns we identified here. Differences might stem from variations in the time points the samples were taken at, the methods applied for methylation analysis and the CpG positions that were actually analyzed. Sampling differences might also play a role in HPV DNA methylation analyses, since cervical swabs often contain diverse cell types and material from multiple lesions as compared to biopsy material. Another difference is that comparable studies used pyrosequencing for analysis. However, a recent report that compared next‐generation‐ and pyrosequencing suggests that the results are similar but not identical with generally higher methylation levels identified by next‐generation sequencing.^50^

With this prospective study, we observed that methylation at the CpG site at nt 5962 located within the *L1* ORF might be predictive for a future persistent HPV infection. Furthermore, we found modest correlations between the methylation of specific CpGs and a high viral load (Table 2). In summary, we demonstrate the potential of using a specific HPV DNA methylation site as biomarker for the determination of a persistent HPV16 infection as risk factor for the development of cervical disease.

**Table 2:** Correlation of viral load with methylation of the viral genome. The methylation for each CpG was correlated with viral load using a Spearman nonparametric correlation (two‐tailed). Only those positions that resulted in a significant correlation (*P * < .05) are shown

	Viral load vs. 494	Viral load vs. 502	Viral load vs. 539	Viral load vs. 5616	Viral load vs. 7033
Spearman r	0.2332	0.2925	0.2448	0.4407	0.399
95% confidence interval	0.03159 – 0.4165	0.09398 – 0.4686	0.04284 – 0.4276	0.1043 – 0.6866	0.09898 – 0.6325
*P* (two‐tailed)	0.0202	0.0035	0.0151	0.0103	0.0089
Exact/approx. *P* value?	approximate	approximate	approximate	approximate	approximate
Number of XY pairs	99	98	98	33	42

## CONFLICT OF INTEREST

CM has received support for conference participation and speaker’s fees from Sanofi Pasteur MSD. SKK has received speaker’s and advisory board fees and research grants through her institution from Sanofi Pasteur MSD and Merck. TI received speaker honoraria from Hologic GmbH, Becton Dickinson Diagnostics GmbH and Sanofi Pasteur MSD; and his host institution (UKT Tuebingen) received an unconditional research grant from Hologic GmbH and Becton Dickinson Diagnostics GmbH.

## Data Availability

The data that support the findings of this study are available from the corresponding author upon reasonable request.

## References

[1] Cogliano V , Baan R , Straif K , et al. Carcinogenicity of human papillomaviruses. Lancet Oncol. 2005;6(4):204. 15830458 10.1016/s1470-2045(05)70086-3

[2] Schiffman M , Wentzensen N , Wacholder S , Kinney W , Gage JC , Castle PE . Human papillomavirus testing in the prevention of cervical cancer. J Natl Cancer Inst. 2011;103(5):368‐383. 21282563 10.1093/jnci/djq562 PMC3046952

[3] McCredie MR , Sharples KJ , Paul C , et al. Natural history of cervical neoplasia and risk of invasive cancer in women with cervical intraepithelial neoplasia 3: a retrospective cohort study. Lancet Oncol. 2008;9(5):425‐434. 18407790 10.1016/S1470-2045(08)70103-7

[4] Doorbar J . Papillomavirus life cycle organization and biomarker selection. Dis Markers. 2007;23(4):297‐313. 17627064 10.1155/2007/613150 PMC3851388

[5] Hoelzer K , Shackelton LA , Parrish CR . Presence and role of cytosine methylation in DNA viruses of animals. Nucleic Acids Res. 2008;36(9):2825‐2837. 18367473 10.1093/nar/gkn121 PMC2396429

[6] Badal V , Chuang LS , Tan EH , et al. CpG methylation of human papillomavirus type 16 DNA in cervical cancer cell lines and in clinical specimens: genomic hypomethylation correlates with carcinogenic progression. J Virol. 2003;77(11):6227‐6234. 12743279 10.1128/JVI.77.11.6227-6234.2003 PMC154984

[7] Bergman Y , Cedar H . DNA methylation dynamics in health and disease. Nat Struct Mol Biol. 2013;20(3):274‐281. 23463312 10.1038/nsmb.2518

[8] Kalantari M , Villa LL , Calleja‐Macias IE , Bernard HU . Human papillomavirus‐16 and ‐18 in penile carcinomas: DNA methylation, chromosomal recombination and genomic variation. Int J Cancer. 2008;123(8):1832‐1840. 18688866 10.1002/ijc.23707 PMC2750853

[9] Kim K , Garner‐Hamrick PA , Fisher C , Lee D , Lambert PF . Methylation patterns of papillomavirus DNA, its influence on E2 function, and implications in viral infection. J Virol. 2003;77(23):12450‐12459. 14610169 10.1128/JVI.77.23.12450-12459.2003 PMC262585

[10] Schubeler D . Function and information content of DNA methylation. Nature. 2015;517(7534):321‐326. 25592537 10.1038/nature14192

[11] Badal S , Badal V , Calleja‐Macias IE , et al. The human papillomavirus‐18 genome is efficiently targeted by cellular DNA methylation. Virology. 2004;324(2):483‐492. 15207633 10.1016/j.virol.2004.04.002

[12] Sugawara K , Fujinaga K , Yamashita T , Ito Y . Integration and methylation of shope papilloma‐virus DNA in the transplantable Vx2 and Vx7 rabbit carcinomas. Virology. 1983;131(1):88‐99. 6316657 10.1016/0042-6822(83)90536-6

[13] Vinokurova S , von Knebel DM . Differential methylation of the HPV 16 upstream regulatory region during epithelial differentiation and neoplastic transformation. PLoS ONE. 2011;6(9):e24451. 21915330 10.1371/journal.pone.0024451 PMC3168499

[14] Bhattacharjee B , Sengupta S . CpG methylation of HPV 16 LCR at E2 binding site proximal to P97 is associated with cervical cancer in presence of intact E2. Virology. 2006;354(2):280‐285. 16905170 10.1016/j.virol.2006.06.018

[15] Brandsma JL , Sun Y , Lizardi PM , et al. Distinct human papillomavirus type 16 methylomes in cervical cells at different stages of premalignancy. Virology. 2009;389(1–2):100‐107. 19443004 10.1016/j.virol.2009.03.029 PMC2918277

[16] Kalantari M , Calleja‐Macias IE , Tewari D , et al. Conserved methylation patterns of human papillomavirus type 16 DNA in asymptomatic infection and cervical neoplasia. J Virol. 2004;78(23):12762‐12772. 15542628 10.1128/JVI.78.23.12762-12772.2004 PMC525027

[17] Manawapat A , Stubenrauch F , Russ R , Munk C , Kjaer SK , Iftner T . Physical state and viral load as predictive biomarkers for persistence and progression of HPV16‐positive cervical lesions: results from a population based long‐term prospective cohort study. Am J Cancer Res. 2012;2(2):192‐203. 22432058 PMC3304573

[18] Human papillomaviruses. IARC Monogr Eval Carcinog Risks Hum. 2007;90:1‐636. 18354839 PMC4781057

[19] Manawapat‐Klopfer A , Wang L , Haedicke‐Jarboui J , et al. HPV16 viral load and physical state measurement as a potential immediate triage strategy for HR‐HPV‐infected women: a study in 644 women with single HPV16 infections. Am J Cancer Res. 2018;8(4):715‐722. 29736316 PMC5934561

[20] Mirabello L , Schiffman M , Ghosh A , et al. Elevated methylation of HPV16 DNA is associated with the development of high grade cervical intraepithelial neoplasia. Int J Cancer. 2013;132(6):1412‐1422. 22847263 10.1002/ijc.27750 PMC3493709

[21] Lillsunde Larsson G , Helenius G , Sorbe B , Karlsson MG . Viral load, integration and methylation of E2BS3 and 4 in human papilloma virus (HPV) 16‐positive vaginal and vulvar carcinomas. PLoS ONE. 2014;9(11):e112839. 25393237 10.1371/journal.pone.0112839 PMC4231157

[22] Kjaer SK , Frederiksen K , Munk C , Iftner T . Long‐term absolute risk of cervical intraepithelial neoplasia grade 3 or worse following human papillomavirus infection: role of persistence. J Natl Cancer Inst. 2010;102(19):1478‐1488. 20841605 10.1093/jnci/djq356 PMC2950170

[23] Boehmer G , Wang L , Iftner A , et al. A population‐based observational study comparing Cervista and hybrid capture 2 methods: improved relative specificity of the Cervista assay by increasing its cut‐off. BMC Infect Dis. 2014;14(1):674. 25487281 10.1186/s12879-014-0674-1 PMC4279999

[24] Petry KU , Luyten A , Justus A , et al. Prevalence of low‐risk HPV types and genital warts in women born 1988/89 or 1983/84 ‐results of WOLVES, a population‐based epidemiological study in Wolfsburg, Germany. BMC Infect Dis. 2012;12:367. 23259726 10.1186/1471-2334-12-367 PMC3536688

[25] Peitsaro P , Johansson B , Syrjanen S . Integrated human papillomavirus type 16 is frequently found in cervical cancer precursors as demonstrated by a novel quantitative real‐time PCR technique. J Clin Microbiol. 2002;40(3):886‐891. 11880410 10.1128/JCM.40.3.886-891.2002 PMC120275

[26] Becker C , Hagmann J , Muller J , et al. Spontaneous epigenetic variation in the Arabidopsis thaliana methylome. Nature. 2011;480(7376):245‐249. 22057020 10.1038/nature10555

[27] Holm S . A simple sequentially rejective multiple test procedure. Scand J Stat. 1979;6(2):65‐70.

[28] Baker CC , Phelps WC , Lindgren V , Braun MJ , Gonda MA , Howley PM . Structural and transcriptional analysis of human papillomavirus type 16 sequences in cervical carcinoma cell lines. J Virol. 1987;61(4):962‐971. 3029430 10.1128/jvi.61.4.962-971.1987 PMC254051

[29] Bauer‐Hofmann R , Borghouts C , Auvinen E , Bourda E , Rosl F , Alonso A . Genomic cloning and characterization of the nonoccupied allele corresponding to the integration site of human papillomavirus type 16 DNA in the cervical cancer cell line SiHa. Virology. 1996;217(1):33‐41. 8599218 10.1006/viro.1996.0090

[30] Sun C , Reimers LL , Burk RD . Methylation of HPV16 genome CpG sites is associated with cervix precancer and cancer. Gynecol Oncol. 2011;121(1):59‐63. 21306759 10.1016/j.ygyno.2011.01.013 PMC3062667

[31] Clarke MA , Wentzensen N , Mirabello L , et al. Human papillomavirus DNA methylation as a potential biomarker for cervical cancer. Cancer Epidemiol Biomarkers Prev. 2012;21(12):2125‐2137. 23035178 10.1158/1055-9965.EPI-12-0905 PMC3664203

[32] Manawapat‐Klopfer A , Thomsen LT , Martus P , et al. TMEM45A, SERPINB5 and p16INK4A transcript levels are predictive for development of high‐grade cervical lesions. Am J Cancer Res. 2016;6(7):1524‐1536. 27508094 PMC4969401

[33] Fernandez AF , Rosales C , Lopez‐Nieva P , et al. The dynamic DNA methylomes of double‐stranded DNA viruses associated with human cancer. Genome Res. 2009;19(3):438‐451. 19208682 10.1101/gr.083550.108 PMC2661803

[34] Lorincz AT , Brentnall AR , Vasiljevic N , et al. HPV16 L1 and L2 DNA methylation predicts high‐grade cervical intraepithelial neoplasia in women with mildly abnormal cervical cytology. Int J Cancer. 2013;133(3):637‐644. 23335178 10.1002/ijc.28050 PMC3708123

[35] Mirabello L , Sun C , Ghosh A , et al. Methylation of human papillomavirus type 16 genome and risk of cervical precancer in a Costa Rican population. J Natl Cancer Inst. 2012;104(7):556‐565. 22448030 10.1093/jnci/djs135 PMC3317880

[36] Wentzensen N , Sun C , Ghosh A , et al. Methylation of HPV18, HPV31, and HPV45 genomes and cervical intraepithelial neoplasia grade 3. J Natl Cancer Inst. 2012;104(22):1738‐1749. 23093560 10.1093/jnci/djs425 PMC3571257

[37] Simanaviciene V , Popendikyte V , Gudleviciene Z , Zvirbliene A . Different DNA methylation pattern of HPV16, HPV18 and HPV51 genomes in asymptomatic HPV infection as compared to cervical neoplasia. Virology. 2015;484:227‐233. 26119875 10.1016/j.virol.2015.06.008

[38] Louvanto K , Franco EL , Ramanakumar AV , et al. Methylation of viral and host genes and severity of cervical lesions associated with human papillomavirus type 16. Int J Cancer. 2015;136(6):E638‐E645. 25203794 10.1002/ijc.29196

[39] Bryant D , Tristram A , Liloglou T , Hibbitts S , Fiander A , Powell N . Quantitative measurement of human papillomavirus type 16 L1/L2 DNA methylation correlates with cervical disease grade. J Clin Virol. 2014;59(1):24‐29. 24268385 10.1016/j.jcv.2013.10.029

[40] Ding DC , Chiang MH , Lai HC , Hsiung CA , Hsieh CY , Chu TY . Methylation of the long control region of HPV16 is related to the severity of cervical neoplasia. Eur J Obstet Gynecol Reprod Biol. 2009;147(2):215‐220. 19819061 10.1016/j.ejogrb.2009.08.023

[41] Kottaridi C , Kyrgiou M , Pouliakis A , et al. Quantitative measurement of L1 human papillomavirus type 16 methylation for the prediction of preinvasive and invasive cervical disease. J Infect Dis. 2017;215(5):764‐771. 28170039 10.1093/infdis/jiw645

[42] Hublarova P , Hrstka R , Rotterova P , et al. Prediction of human papillomavirus 16 e6 gene expression and cervical intraepithelial neoplasia progression by methylation status. Int J Gynecol Cancer. 2009;19(3):321‐325. 19407553 10.1111/IGC.0b013e31819d8a5c

[43] Patel DA , Rozek LS , Colacino JA , et al. Patterns of cellular and HPV 16 methylation as biomarkers for cervical neoplasia. J Virol Methods. 2012;184(1–2):84‐92. 22664184 10.1016/j.jviromet.2012.05.022 PMC3396790

[44] Piyathilake CJ , Macaluso M , Alvarez RD , et al. A higher degree of methylation of the HPV 16 E6 gene is associated with a lower likelihood of being diagnosed with cervical intraepithelial neoplasia. Cancer. 2011;117(5):957‐963. 20945322 10.1002/cncr.25511 PMC3023831

[45] Chaiwongkot A , Niruthisard S , Kitkumthorn N , Bhattarakosol P . Quantitative methylation analysis of human papillomavirus 16L1 gene reveals potential biomarker for cervical cancer progression. Diagn Microbiol Infect Dis. 2017;89(4):265‐270. 28985972 10.1016/j.diagmicrobio.2017.08.010

[46] Liu L , Ying C , Zhao Z , et al. Identification of reliable biomarkers of human papillomavirus 16 methylation in cervical lesions based on integration status using high‐resolution melting analysis. Clin Epigenetics. 2018;10:10. 29410710 10.1186/s13148-018-0445-8 PMC5781301

[47] Torres‐Rojas FI , Alarcon‐Romero LDC , Leyva‐Vazquez MA , et al. Methylation of the L1 gene and integration of human papillomavirus 16 and 18 in cervical carcinoma and premalignant lesions. Oncol Lett. 2018;15(2):2278‐2286. 29434935 10.3892/ol.2017.7596 PMC5776931

[48] Hsu YW , Huang RL , Su PH , et al. Genotype‐specific methylation of HPV in cervical intraepithelial neoplasia. J Gynecol Oncol. 2017;28(4):e56. 28541643 10.3802/jgo.2017.28.e56 PMC5447154

[49] Gillio‐Tos A , Fiano V , Grasso C , et al. Assessment of viral methylation levels for high risk HPV types by newly designed consensus primers PCR and pyrosequencing. PLoS ONE. 2018;13(3):e0194619. 29579066 10.1371/journal.pone.0194619 PMC5868804

[50] Mirabello L , Frimer M , Harari A , et al. HPV16 methyl‐haplotypes determined by a novel next‐generation sequencing method are associated with cervical precancer. Int J Cancer. 2015;136(4):E146‐E153. 25081507 10.1002/ijc.29119 PMC4262737

