# Supplementary material for: Methylation of CpG 5962 in L1 of the human papillomavirus 16 genome as a potential predictive marker for viral persistence: A prospective large cohort study using cervical swab samples
Source: Cancer Med. 2019 Dec 19;9(3):1058–68. doi: 10.1002/cam4.2771 (PMC6997067; doi:10.1002/cam4.2771)
Supplement: Supplementary file 1 [file CAM4-9-1058-s001.pdf]

Supplemental Material

Supplemental Table S1: Primers used in this study.

Name	Sequence 5`-3`	Amplicon length
BS1 7893-7916 for	ATGAATTGTGTAAAGGTTAGTTAT	
BS1 101-123 rev	TGTAATGTTTTAGGATTTATAGGA	233
BS2 84-107 for	TGTATTAAAAGAGAATTGTAATGT	
BS2 355-380 rev	TGGAATAATATTAGAATAGTAATATA	297
BS3 292-313 for	TGATAAATGTTTAAAGTTTTAT	
BS3 463-492 rev	TAAAGATTTTATAATATAAGGGGT	202
BS4 404-428 for	ATTAGGTGTATTAATTGTTAAAAGT	
BS4 655-676 rev	TTAGAGGAGGAGGATGAAATAG	273
BS5 565-592 for	TATGGAGATATATTTATATTGTATG	
BS5 865-892 rev	ATGGTTGATTTTGTAGGTATTAATGG	326
BS6 5594-5615 for	TAGTTTTAGGGTTTTTATAATA	
BS6 5816-5840 rev	GTAGGAATATTTAGATTATTTGTAG	246
BS7 5815-5837 for	TGTAGGAATATTTAGATTATTTG	
BS7 6111-6133 rev	ATGTAGTAAATGTAGGTGTGGAT	315
BS8 6323-6343 for	GGTGTTATGGATTTTATTATA	
BS8 6671-6695 rev	GGTATTTGTTGGGGTAATTAATTAT	373
BS9 6671-6691 for	GGTATTTGTTGGGGTAATTAA	
BS9 6938-6959 rev	GGAGGTATATTAGAAGATATTT	289
BS10 7079-7101 for	GTAGATTTAGATTAGTTTTTTTT	
BS10 7448-7468 rev	GTGTTGTGGTTATTTATTGTA	372
BS11 7448-7470 for	GTGTTGTGGTTATTTATTGTATA	
BS11 7670-7692 rev	TGATTTGTATTGTTTGTTAATTA	243
BS12 7671-7694 for	GATTTGTATTGTTTGTTAATTATT	
BS12 7796-7816 rev	TAGGTATATATTTTTGGTTTG	144
M13 for	GTAAACGACGGCCAGTG	
M13 rev	GGAAACAGCTATACCATGA	

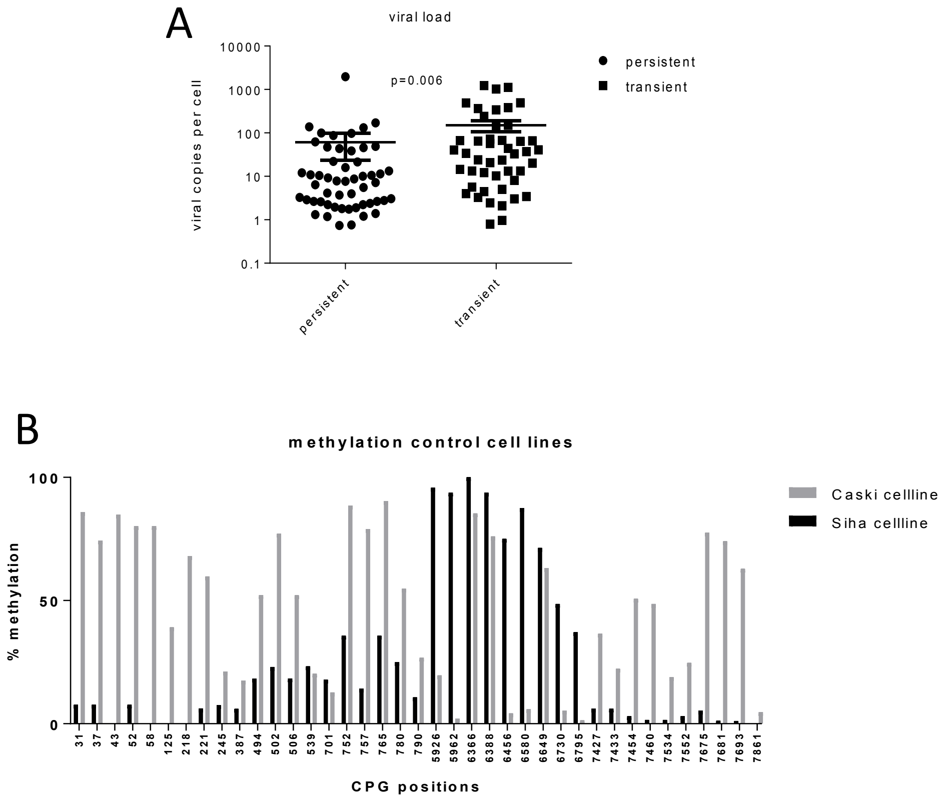

Supplemental Figure S1:

A: Viral loads in the analyzed samples. The samples were analyzed by quantitative Real Time PCR using primers for E6 and a single copy gene Ifnb. The samples were grouped according to their future infection outcome and a Mann Whitney test was used to test for statistical significance.

B: The methylation at each CpG for each cell line was calculated, (each bar represents the mean value)

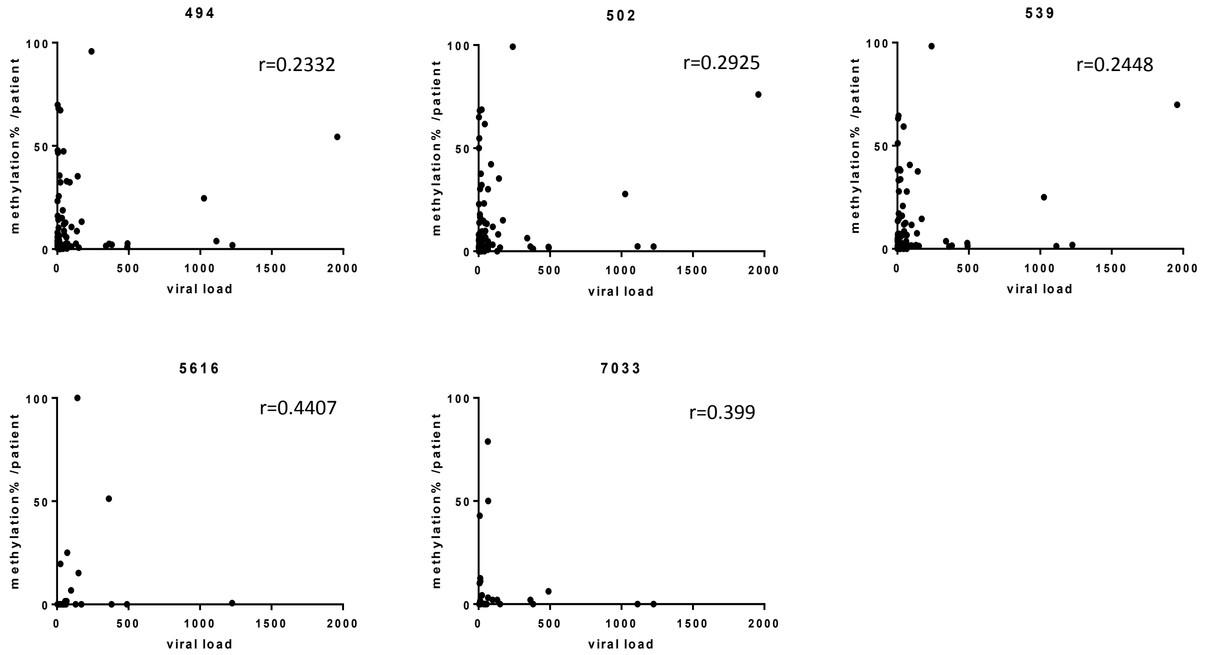

Supplemental Figure S2:

Correlation graphs of methylation percentage per patient with viral load for the significantly tested CpGs in Table 2. Viral load is plotted on the x-axis, while methylation in percent is plotted on the y-axis. Each dot represents one patient. r=Pearson's correlation coefficient
